# Risk of Adverse Pregnancy Outcomes in Patients with Non-Communicable, Chronic Inflammatory Barrier Diseases Under Systemic Treatment: A Large-Scale Retrospective Cohort Study

**DOI:** 10.3390/biom16081083

**Published:** 2026-07-24

**Authors:** Inga Catharina Brouer, Aida Zani, Philip Curman, Ralf J. Ludwig, Diamant Thaçi

**Affiliations:** 1 Institute and Comprehensive Center for Inflammation Medicine, University of Lübeck, 23562 Lübeck, Germany; aida.zani@uksh.de (A.Z.); diamant.thaci@uksh.de (D.T.); 2 Lübeck Institute of Experimental Dermatology, University of Lübeck, 23562 Lübeck, Germanyralf.ludwig@uksh.de (R.J.L.); 3 Department of Medical Epidemiology and Biostatistics, Karolinska Institutet, 17177 Stockholm, Sweden; 4 Dermatology and Venereology Division, Department of Medicine (Solna), Karolinska Institutet, 17177 Stockholm, Sweden; 5 Dermato-Venereology Clinic, Karolinska University Hospital, 17176 Stockholm, Sweden; 6 Department of Dermatology, University Medical Centre of the State of Schleswig-Holstein (UKSH), 23538 Lübeck, Germany

**Keywords:** chronic inflammatory barrier diseases, pregnancy, adverse pregnancy outcomes, TNF inhibitors, IL-23 inhibitors, csDMARDs

## Abstract

Chronic inflammatory barrier diseases (CIBDs) affect many women of reproductive age, yet the safety of biologics during pregnancy remains inadequately investigated. This retrospective cohort study used the US Collaborative Network of TriNetX to evaluate the risk of adverse pregnancy outcomes (APOs) among women with CIBD receiving tumor necrosis factor inhibitors (TNFis), interleukin-23 inhibitors (IL-23is), or conventional synthetic disease-modifying anti-rheumatic drugs (csDMARDs), compared with untreated CIBD controls and the general pregnant population. Among 1842 pregnant women with CIBD receiving systemic therapy, 1188 were exposed to TNFis, 170 to IL-23is, 370 to azathioprine (csDMARD), and 114 to methotrexate (MTX) (csDMARD). The incidence of any APO ranged from 11% in untreated CIBD controls to 19% with ustekinumab (IL-12/23i). Among treatment groups, rates were 17% with TNFis, 18% with IL-23is, 13% with azathioprine, and 15% with MTX, compared with 17% in the general pregnant population. APO rates were comparable between the overall TNFi group and the TNFi group excluding certolizumab pegol (CZP) (TNFi). In the only non-exploratory comparison, TNFi exposure was associated with higher risks of (pre-)eclampsia and hypertension but a lower risk of abortion or intrauterine death versus CIBD controls, with no significant difference for overall APO. No treatment group showed a markedly increased overall APO risk, supporting tailored decision-making that balances the consequences of uncontrolled maternal disease against individual treatment-associated risks.

## 1. Introduction

Non-communicable, chronic inflammatory barrier diseases (CIBDs) include various immune-mediated inflammatory diseases (IMID) affecting different organ systems and imposing substantial physical, psychosocial, and economic burdens on patients and healthcare systems [1,2]. Common examples of CIBDs are rheumatoid arthritis (RA); Crohn’s disease (CD) and Ulcerative colitis (UC) (inflammatory bowel disease (IBD)); and psoriasis [3]. These conditions are highly prevalent, with psoriasis affecting approximately 2–3% of the global population, RA approximately 0.2–1%, and IBD approximately 0.1–0.12% [2,4,5,6].

As these diseases frequently manifest during the reproductive years, many women of childbearing age require ongoing pharmacological treatment, including biologic therapies. However, most of these treatments are not approved for use during pregnancy, and safety data remain limited and are mainly derived from observational studies and case reports [7].

Treatment continuation during pregnancy varies between diseases. In IBD, continuation of therapy is more common, whereas in RA and psoriasis, treatment is more frequently interrupted [8]. This difference in treatment continuation is clinically important, as disease flares resulting from treatment discontinuation have been associated with adverse pregnancy outcomes (APOs), including preterm birth, low birth weight, and spontaneous abortion [9]. Continued biologic treatment during pregnancy may benefit selected patients by maintaining disease control and reducing maternal and fetal complications. This is supported by case reports of severe, uncontrolled disease, including Crohn’s disease with paradoxical psoriasis, and pustular psoriasis with psoriatic arthritis, suggesting that continuation or re-initiation of biologic therapy during pregnancy may be justified in selected patients [10,11].

A variety of medication classes are available for CIBDs, including conventional disease-modifying antirheumatic drugs (csDMARDs) such as methotrexate (MTX) and azathioprine, biologic agents such as tumor necrosis factor inhibitors (TNFis, including adalimumab, infliximab, golimumab, and certolizumab pegol (CZP)), interleukin-12/23 inhibitors (IL-12/23is; ustekinumab) and interleukin-23 inhibitors (IL-23is, including guselkumab, risankizumab, and tildrakizumab). The use of biologic agents, particularly TNFis and IL-12/23is or IL-23is, in CIBDs are increasing [12], including in pregnant women [13]. Consequently, generating reliable safety data in pregnancy would greatly improve treatment decision-making.

Previous meta-analyses have investigated APOs in pregnant women with CIBDs who received biologic treatment, compared to disease-matched control groups not exposed to biologics [14,15]. One systematic review and meta-analysis including women with RA, IBD, ankylosing spondylitis, and psoriasis/psoriatic arthritis and focusing on maternal and neonatal outcomes found higher crude odds for APOs such as preterm birth, low birth weight, and congenital anomalies [15]. However, after adjustment for potential confounders, including maternal age, disease activity, comorbidities, concomitant medication use, and other relevant demographic and clinical variables, the association with congenital anomalies was no longer statistically significant. Another systematic review and meta-analysis found no significant differences in APOs among patients with rheumatoid arthritis, psoriatic arthritis, and IBD [14]. However, this analysis was based on unadjusted proportions without adjustment for patient-level confounders, increasing the risk of residual confounding. Both studies lacked comparator groups involving alternative treatments. Therefore, it is difficult to distinguish drug effects from baseline disease risk. Concerning IL-23is, data on APOs in patients with CIBDs are limited, with most evidence derived from studies on ustekinumab (p40 inhibitor targeting IL-12 and IL-23). For instance, a narrative review did not detect an increased risk of APOs with ustekinumab exposure in pregnant women with CIBDs including psoriasis, psoriatic arthritis, and IBD. However, it was based on a small number of exposed pregnancies [16]. Data from the PIANO registry, a multicentre prospective observational study that began in 2007, collected data using questionnaires. These were completed during pregnancy and up to one year after delivery. The results showed no increased risk of APOs in pregnant women treated with ustekinumab compared to those receiving TNFis, immunomodulators, or combination therapy. The study focused exclusively on women with IBD [9].

Finally, no large-scale study has systematically analysed APOs across pooled CIBDs and diverse therapeutic classes within a single unified analysis. This gap is particularly relevant for biologics and other conventional systemic drugs, which are largely not approved for use in pregnancy due to the absence of data from randomized controlled trials (RCTs). Conducting RCTs in this setting would be both ethically and logistically challenging, highlighting an area where real-world experience (RWE) data can address questions that RCTs cannot [17]. Our objective was therefore to bridge this gap by providing a comprehensive, real-world descriptive overview of APOs in women with pooled CIBDs, including psoriasis, CD, UC, and RA. This analysis encompassed patients receiving various systemic therapies as well as appropriate control groups, leveraging a large, multicentre electronic health record network (TriNetX). These descriptive findings offer an important reference point for clinicians and researchers, particularly in a domain where high-quality comparative evidence remains scarce.

## 2. Materials and Methods

### 2.1. Ethics Statement

Data retrieved from TriNetX are presented collectively. Only de-identified patient data were used, which waived the need for Institutional Review Board approval or written informed consent. The use of the TriNetX network for this research was approved by the Swedish Ethical Review Authority.

### 2.2. Study Design and Database

**Figure 1:**
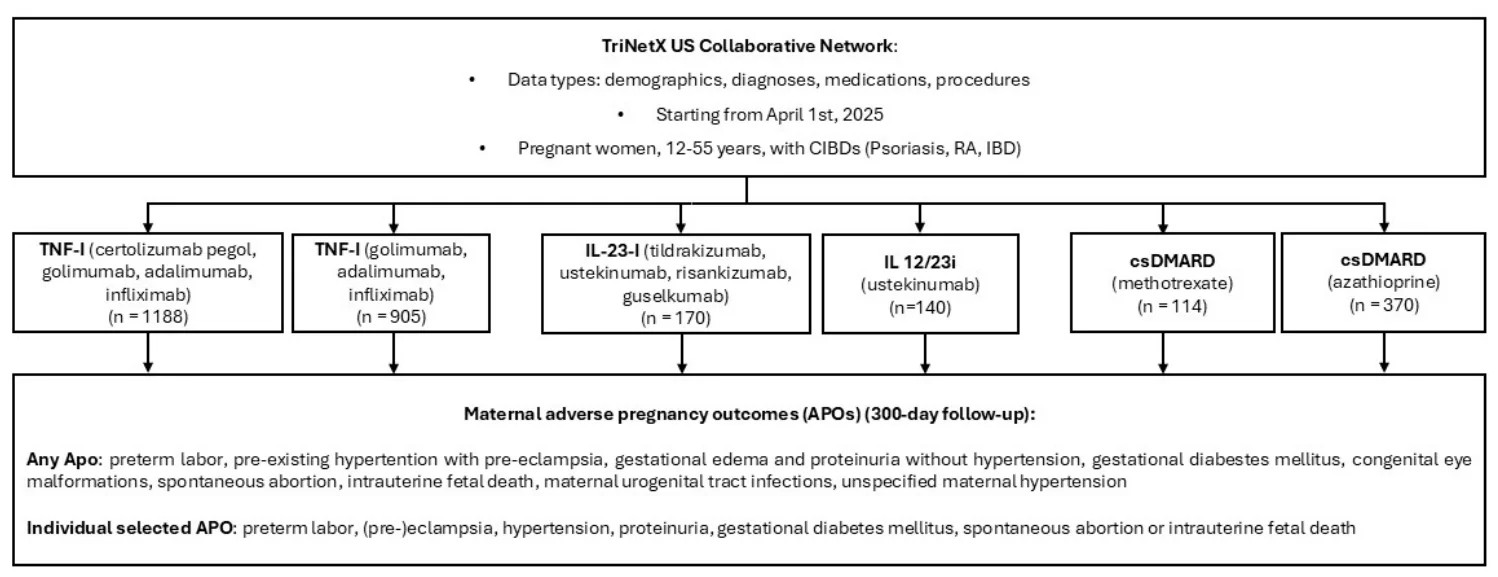
Flowchart of study inclusion. Data were obtained from the TriNetX US Collaborative Network. Pregnant women aged 12–55 years with chronic inflammatory diseases (psoriasis, rheumatoid arthritis, or inflammatory bowel disease) were identified. Patients were categorized according to treatment exposure, including TNF inhibitors, IL-23 inhibitors, IL-12/23 inhibitor therapy, and conventional synthetic disease-modifying antirheumatic drugs (csDMARDs). Maternal adverse pregnancy outcomes (APOs) were assessed during a 300-day follow-up period. APOs included preterm labor, pre-eclampsia, hypertension, proteinuria, gestational diabetes mellitus, spontaneous abortion, intrauterine fetal death, congenital malformations, and maternal infections. CIBD, chronic inflammatory barrier disease; RA, rheumatoid arthritis; IBD, inflammatory bowel disease; TNF, tumor necrosis factor; IL, interleukin; csDMARD, conventional synthetic disease-modifying antirheumatic drug; APO, adverse pregnancy outcome.

A retrospective cohort study was performed using the TriNetX Research Network (TriNetX LLC, Cambridge, MA, USA). TriNetX provides access to de-identified electronic health records (EHRs), including information about demographic data (age, sex), diagnoses (ICD-10-CM), procedures (HCPCS), and medications (RXNORM) [18]. To facilitate this, TriNetX establishes partnerships with healthcare organizations (HCOs), where contributing HCOs have access to the database and analytical pipelines. We retrieved data for our predefined cohorts consisting of pregnant women, both with and without CIBDs, and with or without the respective medications (TNFis, IL-23is, csDMARDs). The data were analyzed within a 300-day follow-up period starting from the index event, which is defined as the initial pregnancy documentation. The collection of the data for this study was performed in May 2025. Figure 1 presents the study design.

### 2.3. Study Population

EHRs from females aged 12 to 55 years were retrieved from the US Collaborative Network of TriNetX. Pregnant women within this population were identified using the International Classification of Diseases (Tenth Revision) ICD-10-CM codes, the Procedure Coding System (ICD-10-PCS) codes, and Current Procedural Terminology (CPT) codes (Supplementary Table S1). To assess the risk of APOs we categorized the EHRs into six study groups and two control groups (Supplementary Table S2). The study groups included patients who had any of the following CIBDs: (1) psoriasis (L40), including psoriatic arthritis; (2) IBD, including CD (K50) or UC (K51.90); and (3) RA, including RA with rheumatoid factor (M05.9) or other types of RA (M06). Patients were also included if they had been treated with any of the following: (1) TNFis [CZP (RxNorm 709271), golimumab (RxNorm 819300), adalimumab (RxNorm 327361), infliximab (RxNorm 191831)], (2) IL-23is [tildrakizumab (RxNorm 2053436), ustekinumab (RxNorm 847083), risankizumab (RxNorm 2166040), guselkumab (RxNorm 1928588)], or (3) csDMARDs [MTX (RxNorm 6851), azathioprine (RxNorm 1256)]. IL-17 inhibitors were excluded due to lack of approval for IBD and RA, as well as the potential risk of disease exacerbation in IBD [19]. JAK inhibitors, apremilast, and TYK2 inhibitors were excluded due to pregnancy safety concerns and insufficient data. To ensure medication exposure during pregnancy, two exposure definitions were applied within TriNetX. In the primary definition, patients were required to have at least one record of the medication on or one day after the index event (onset of pregnancy). To further capture sustained treatment, a secondary definition required an additional record of medication exposure between one and three months following the index event. Only medications with a minimum of 50 patients in the respective cohorts were included in the analysis to ensure sufficient statistical power.

### 2.4. Outcomes

We analyzed the occurrence of the APOs within 300 days after the code indicating pregnancy. We distinguished between ‘any APO’, and individual selected APOs (Supplementary Table S3). Individual selected APOs included preterm labor (O60), (pre-)eclampsia (O11, O14, O15), hypertension (O13, O16), proteinuria (O12), gestational diabetes (O24.4) and spontaneous abortion (O03) and/or intrauterine fetal death (O36.4) as a composite endpoint. Patients with outcomes prior to the index event were excluded from each analysis.

### 2.5. Analyses and Statistics

#### 2.5.1. Descriptive Analysis

Statistical analyses were performed on the TriNetX platform. Continuous variables are summarized as means with their corresponding standard deviations, while categorical variables are reported as counts and percentages. To assess the APO risk for each medication, we conducted a risk analysis, using cumulative incidence, calculated as the proportion of patients with at least one APO during the observation period for each treatment group. In addition, survival data were evaluated through Kaplan–Meier analysis. Three predefined sensitivity analyses were performed to evaluate the robustness of our findings. Sensitivity analysis 1 focused on the risk of APOs in patients with IBD treated with systemic therapies during pregnancy, thereby restricting the population to a specific CIBD subgroup. Sensitivity analysis 2 restricted the cohort to pregnancies in adults aged 18 to 55 years, accounting for potential age-related confounding factors. Sensitivity analysis 3 limited the sample to pregnancies with an index event occurring in 2019 or later, corresponding to the timeframe when all analyzed medications were available on the market.

#### 2.5.2. Comparative Analysis

In addition to the descriptive analysis, a pairwise comparison of selected treatment groups was performed. These analyses included comparisons between (1) TNFis and IL-23is, (2) TNFis and MTX, (3) TNFis and azathioprine, (4) IL-23is and MTX, and (5) IL-23is and azathioprine. Additionally, each treatment group was compared with the CIBD control cohort. For these comparative analyses, propensity score matching (PSM) was used to match the compared groups according to demographic variables (age, ethnicity), socioeconomic and psychosocial circumstances, comorbidities that may affect the risk of APOs (overweight and obesity, hypertensive diseases, nicotine dependence, diabetes mellitus, alcohol-related disorders, chronic kidney disease) and common medications (adrenal corticosteroids, gastric medications, sulfasalazine) (Supplementary Table S7). Continuous variables were analyzed using Student’s *t*-test, and categorical variables were compared using the Pearson chi-square test. Group differences were evaluated using the log-rank test, and hazard ratios (HRs) were derived from Cox proportional hazards models. The Bonferroni correction adjusted the significance level to α = 0.0071 for each of the seven comparisons.

## 3. Results

### 3.1. Study Population

For the descriptive analysis, EHRs of pregnant women diagnosed with a CIBD were evaluated across multiple exposure groups (Table 1):

Patients receiving a TNFi (*n* = 1188), a TNFi excluding CZP (*n* = 905), MTX (*n* = 114), azathioprine (*n* = 370), an IL-23i (*n* = 170), or an IL-12/23i (ustekinumab) (*n* = 140) were included. As control groups, pregnant women in the same age range (12–55 years) also diagnosed with psoriasis, IBD and/or RA and not receiving biologic treatment (*n* = 101,255), as well as all pregnant women aged 12–55 years (*n* = 5,155,718), were included.

The mean age ranged from 30.7 ± 6.15 years (TNFi w/o CZP) to 36.8 ± 8.39 years (MTX) amongst the exposure groups, with the CIBD control group and all pregnant women aged 34.9 ± 8.93 and 28.5 ± 6.59 years, respectively. Most patients in each cohort were White, with proportions ranging from 74.80% (TNFi w/o CZP) to 54.23% (all pregnant women). Black or African American patients comprised between 17.83% (azathioprine) and 8.57% (ustekinumab) of the treatment cohorts. Hispanic or Latino participants were represented across all groups, ranging from 17.32% in the pregnant control group to 7.57% in the azathioprine group. Asian patients accounted for 8.77% (MTX) to 3.09% (TNFi w/o CZP) of the cohorts. The prevalence of alcohol-related disorders ranged from 1.00% (all pregnant women) to 9.00% (MTX). Nicotine dependence showed a similar pattern, with prevalence at 16.00% (MTX) and 3.00% (all pregnant women).

### 3.2. Descriptive Risk of APOs

The following descriptive analysis presents unadjusted cumulative incidence rates to provide an exploratory overview of APO frequencies. Adjusted comparisons using PSM are reported separately in Section 3.3. The incidence of APOs was analyzed separately for each treatment and control group (Table 2). For each cohort, the proportion of patients experiencing any APO as well as the frequencies of specific APOs were determined. The analysis for the exposure groups revealed that the rates of any APO ranged from 19.00% in the ustekinumab group to 13.15% in the azathioprine group. The IL-23i group had an APO rate of 17.95%, followed by the TNFi groups with 17.20% (TNFi) and 16.75% (TNFi w/o CZP). The second control group (all pregnant women) presented an APO rate of 16.63%, while the MTX group had a rate of 15.15%. The CIBD control group had the lowest APO rate at 11.40%.

For (pre-)eclampsia, the highest rate was observed in the MTX group (9.90%), followed by the TNFi group (7.65%), ustekinumab (7.41%), TNFi w/o CZP (6.92%), IL-23i (6.71%), azathioprine (4.46%), all pregnant women (3.77%), and the CIBD control group (2.57%). Regarding abortion or intrauterine fetal death, ustekinumab had the highest rate (7.58%), followed by IL-23i (6.29%), all pregnant women (3.28%), azathioprine (2.98%), CIBD control (2.20%), TNFi w/o CZP (1.44%), TNFi (1.20%), and MTX (0).

To explore the impact of underlying disease heterogeneity, a sensitivity analysis restricted to patients with IBD was performed. In this subgroup, APO rates showed a broadly similar pattern across treatment groups compared to the overall cohort, with variability persisting between exposures. Detailed results of all three sensitivity analyses are provided in Supplementary Tables S4–S6.

### 3.3. Comparative Analyses

Except for TNFi versus CIBD controls, small cohort sizes limited statistical power in the comparative analyses, and the results should therefore be interpreted as exploratory. In these results, TNFi exposure during pregnancy was associated with a significantly increased risk of (pre-)eclampsia (HR 2.07, 95% CI 1.43–3.01, *p* < 0.0001), hypertension (HR 1.97, 95% CI 1.44–2.70, *p* < 0.0001), and proteinuria (HR 2.40, 95% CI 1.26–4.57, *p* = 0.0060) compared to the matched CIBD control group. No significant differences were observed for gestational diabetes mellitus, preterm labor or the composite endpoint of any APO. The risk of abortion or intrauterine death was significantly lower with TNFis compared to the matched CIBD control group (HR 0.39, 95% CI 0.21–0.74, *p* = 0.0029) (Supplementary Table S8). For all other drug comparisons, no statistically significant differences were detected, likely due to insufficient sample size and power.

## 4. Discussion

Our study cohort included 1842 pregnant women with CIBDs, all of whom were of reproductive age (12–55 years), with mean age at index ranging from 30.7 years (TNFi w/o CZP) to 36.8 years (MTX). The higher age for MTX users likely reflects its teratogenic risk, which requires effective contraception and a washout period before conception [20]. Pregnancies under MTX may therefore be more frequently unplanned, particularly in older women with less rigorous contraceptive practices or those who initiated MTX before biologic agents became widely available [21,22]. Compared to the population-based control group (mean age 28.5 years), most CIBD cohorts were older at conception, possibly reflecting delayed childbearing due to disease burden and treatment requirements.

In this exploratory, real-world analysis of APOs across multiple systemic therapies for CIBDs, we observed broadly reassuring patterns. When considering the composite endpoint of “any APO,” rates were remarkably similar across therapeutic classes and largely correspond to those of the general control group of pregnant women. Overall, these findings do not indicate a clear signal of increased maternal APO rates associated with systemic treatment in this cohort. To address potential confounding by indication across different CIBD subtypes, we performed a sensitivity analysis restricted to IBD. While this approach reduced disease heterogeneity, variability in APO rates across treatment groups persisted. These findings suggest that the differences observed in the main analysis are unlikely to be solely explained by disease composition. However, residual confounding related to disease severity and differences in pregnancy management cannot be excluded. Although stratification by individual CIBD would be desirable, sample sizes for the RA and psoriasis subgroups were insufficient to permit meaningful disease-specific analyses beyond the IBD sensitivity analysis already performed. This remains an important interpretive constraint, as each disease carries distinct baseline APO risk profiles and treatment patterns that may differentially influence the observed outcomes. Among women in our cohort who were exposed to azathioprine, the incidence of APOs was generally low, the lowest among all treatment groups, and comparable to the CIBD control group. Rates of individual APOs, including (pre-)eclampsia (4.46%), preterm labour (4.62%), and gestational diabetes (2.96%), did not suggest a clinically meaningful increase in risk. These findings are consistent with the existing literature. Azathioprine is FDA-approved for the management of RA and is widely used off-label in IBD and psoriasis. The 2020 American College of Rheumatology (ACR) Guideline for the management of reproductive health in rheumatic and musculoskeletal diseases recommends the continuation of azathioprine throughout pregnancy, reflecting its favourable safety profile relative to other immunosuppressants [23]. Similarly, the American College of Obstetricians and Gynecologists (ACOG) states that existing data do not demonstrate azathioprine to be a human teratogen, although a few reports suggest an increased risk of preterm birth and fetal growth restriction [24]. A systematic review that included over 300 pregnancies compared APOs in women with IBD exposed to thiopurines like azathioprine to those on other IBD medications or no medication [25]. The analysis found no significant differences between thiopurines and other IBD drugs for prematurity, low birth weight, congenital abnormalities, spontaneous abortion, or neonatal adverse outcomes. However, compared to no medication, thiopurines were associated with a significantly increased risk of congenital abnormalities, while other outcomes showed no significant differences. Taken together, both our data and the published evidence suggest that azathioprine is not associated with a substantial increase in the risk of APOs [26].

In our cohort, the incidence of APOs among women exposed to MTX was 15.15%. Given the small sample size, our data are insufficient to draw independent conclusions regarding APO risk with this agent. These observations must be interpreted in the context of the established teratogenicity of MTX. The ACR strongly recommends discontinuation of MTX at least three months prior to conception, and the FDA label classifies MTX as contraindicated in pregnant women with non-neoplastic diseases due to its known abortogenic and teratogenic effects [23,27]. There is limited published data specifically addressing maternal APOs associated with MTX use during pregnancy. A prospective multicentre cohort study evaluated pregnancy outcomes in women taking MTX ≤30 mg per week after conception [28]. The cumulative incidence of spontaneous abortion in the post-conception exposure cohort was 42.5% (95% CI 29.2–58.7), which was significantly higher than in disease-matched unexposed patients (22.5%, 95% CI 16.8–29.7) and unexposed patients without autoimmune disease (17.3%, 95% CI 13.0–22.8). The risk of major birth defects was also elevated at 6.6% compared with 2.9% in women without autoimmune diseases (adjusted OR 3.1, 95% CI 1.03–9.5), although none of the malformations were clearly consistent with MTX embryopathy.

In our cohort, the overall incidence of any APO among TNFi-exposed pregnancies was comparable to the general pregnant population but higher than the disease-matched unexposed control group. APO rates were similar between the overall TNFi group and the subgroup excluding CZP (16.75%). The comparative analysis showed significantly increased HRs for (pre-)eclampsia, hypertension, and proteinuria versus CIBD controls, while the composite APO endpoint, gestational diabetes, and preterm labour did not differ significantly. The risk of spontaneous abortion and/or intrauterine fetal death was significantly lower in the TNFi group. These rates may partly reflect underlying systemic inflammation and confounding by indication, although a direct pharmacological effect cannot be excluded based on these observational data. These findings align with the broader literature.

Most TNFi agents cross the placenta, especially in late pregnancy, potentially affecting the fetal immune system and increasing the risk of APOs [29]. CZP, lacking an Fc region, shows minimal placental transfer and was therefore also analyzed separately from other TNFis due to its distinct pharmacokinetic profile [23]. The 2020 ACR Guideline recommends continuing TNFis during pregnancy and strongly recommends continuation of CZP given its minimal placental transfer. The safety of TNFis during pregnancy has been assessed across multiple IMIDs, although most published data focus on neonatal outcomes and congenital malformations rather than maternal APOs. A systematic review and meta-analysis of 39 studies across IBD, RA, and psoriasis found an increased risk of preterm birth (OR 1.45, 95% CI 1.16–1.82) in TNFi-exposed women compared with diseased controls. When stratified by disease, this risk was driven primarily by IBD patients, whereas RA and psoriasis patients showed no elevated risk [30]. Data from the PIANO Registry provide stronger evidence supporting safety. Involving 1490 pregnant women with IBD, the registry evaluated the safety of in utero exposure to biologics. Among 1431 live births and 1-year follow-up data from 1010 infants, exposure to biologic therapy was not associated with an increased risk of APOs, including preterm birth, spontaneous abortion, low birth weight, congenital malformations, or infant infections. Instead, higher maternal disease activity was independently linked to increased risks of spontaneous abortion, preterm birth, and infant infections [31]. These findings support the continued use of TNF-targeted therapy during pregnancy to maintain disease control and reduce the likelihood of adverse maternal and neonatal outcomes.

In our cohort, the incidence of any APO was comparable between the overall IL-23i group and the general pregnant population. IL-23is are a recent class of monoclonal antibodies that have demonstrated high efficacy in the treatment of psoriasis and IBD [32,33]. However, experience with their use during pregnancy remains limited, and available safety data are derived mainly from case series, registry data, and small cohort studies. Recent findings from a WHO global pharmacovigilance study (1968–2024) support the relative safety of these agents during pregnancy in psoriasis, with most IL-23is showing lower reporting odds ratios for APOs compared with TNFis [34]. Specifically, ustekinumab (ROR 0.27), guselkumab (0.09), and tildrakizumab (0.02) demonstrated lower frequencies of APOs, although risankizumab, despite an overall lower risk (0.38), was associated with a higher rate of abortion and stillbirth (ROR 1.87).

In addition to the overall IL-23i analysis, ustekinumab was analysed separately, as its simultaneous inhibition of both IL-12 and IL-23 distinguishes it mechanistically and clinically [35]. The numerically higher APO rate in the ustekinumab subgroup could hypothetically relate to its concurrent IL-12 blockade. IL-12 suppresses TNF-α production at the transcriptional level, and its inhibition may theoretically lead to unopposed TNF-α activity at the maternal–fetal interface [36]. While elevated TNF-α has been linked to pre-eclampsia, gestational hypertension, and pregnancy loss [37], this mechanistic hypothesis remains speculative and requires validation in dedicated studies. Selective IL-23p19 inhibitors preserve this IL-12-mediated regulation, potentially explaining their more favourable maternal safety profile.

While these findings provide important insights, several limitations must be considered. Our analysis did not stratify APO rates by underlying disease entity (RA, psoriasis, IBD). Published data demonstrate that each CIBD carries a distinct APO risk profile independent of treatment [38]. To address this potential confounding by indication, we performed a sensitivity analysis restricted to IBD patients. While this reduced disease heterogeneity, variability in APO rates across treatment groups persisted, suggesting that the differences observed in the main analysis are unlikely to be solely explained by disease composition.

In addition, treatment continuation during pregnancy varies by disease, with biologic therapy more often continued in IBD compared with psoriasis or RA. To mitigate this potential confounding, we designed the cohort to include only patients with documented biologic exposure for at least 1 to 3 months during pregnancy.

Further limitations relate to the retrospective EHR-based design. Reliance on ICD-coded outcomes may introduce misclassification or underreporting. Only patients with documented encounters within TriNetX-affiliated healthcare systems were captured, potentially introducing selection bias. Sample sizes varied considerably, particularly in the MTX and ustekinumab groups, limiting statistical power for subgroup comparisons. Data on medication dose and adherence were unavailable, so the results should be interpreted as intention-to-treat. Moreover, trimester-specific stratification of drug exposure was not feasible due to the lack of reliable gestational age data within the TriNetX platform; this remains an important objective for future prospective studies. The absence of disease activity data is a further constraint, as untreated patients may have had more active disease, confounding the observed associations. Finally, the analysis focused on maternal APOs, and limited information on fetal or neonatal outcomes restricts conclusions regarding the safety of these therapies for the child.

As obtaining prospective data is challenging in this context, our study provides an important foundation of RWE regarding the safety of biologic treatment during pregnancy in women with CIBD and provides a valuable basis for clinical decision-making in this vulnerable patient population.

## 5. Conclusions

In this large-scale retrospective cohort study, systemic treatment of CIBDs during pregnancy was not associated with a clinically relevant increase in overall APOs. These findings support individualized treatment decisions that consider both the potential risks of therapy and the consequences of uncontrolled maternal disease. Further prospective studies are needed to validate these observations and better define treatment-specific safety profiles.

## Figures and Tables

**Table 1:** Baseline characteristics of the study cohort. Continuous variables are presented as mean ± standard deviation (SD) and range (minimum–maximum), while categorical variables are presented as number (n) and percentage (%). TNFis, tumor necrosis factor inhibitors; CZP, certolizumab pegol; MTX, methotrexate; IL-23is, interleukin-23 inhibitors; CIBD, chronic inflammatory barrier disease; SD, standard deviation.

Characteristic	Subgroup	TNFis	TNFis (w/o CZP)	MTX	Azathioprine	IL-23is	Ustekinumab	CIBD Control	Control Pregnant Women
N (Total)		1188	905	114	370	170	140	101,255	5,155,718
Age at Index (years)	Mean ± SD	31.1 ± 6.09	30.7 ± 6.15	36.8 ± 8.39	32 ± 5.85	32 ± 6.02	31.7 ± 5.51	34.9 ± 8.93	28.5 ± 6.59
	Min–Max	15–58	15–58	19–55	19–54	17–52	20–49	12–86	12–55
Sex	Female	1188 (100%)	905 (100%)	114 (100%)	370 (100%)	170 (100%)	140 (100%)	101,255 (100%)	5,155,718 (100%)
Ethnicity	Not Hispanic or Latino	882 (74.24%)	685 (75.69%)	85 (74.56%)	274 (74.05%)	133 (78.23%)	108 (77.14%)	70,687 (69.81%)	2,838,328 (55.05%)
	Hispanic or Latino	112 (9.43%)	70 (7.74%)	15 (13.16%)	28 (7.57%)	14 (8.24%)	11 (7.86%)	11,447 (11.31%)	892,784 (17.32%)
	Unknown Ethnicity	194 (16.33%)	150 (16.57%)	14 (12.28%)	68 (18.38%)	23 (13.53%)	21 (15%)	19,121 (18.88%)	1,424,806 (27.63%)
Heritage	White	886 (74.57%)	677 (74.80%)	75 (65.78%)	250 (67.56%)	127 (74.70%)	102 (72.85%)	71,774 (70.88%)	2,795,596 (54.23%)
	Black or African American	145 (12.20%)	120 (13.25%)	15 (13.15%)	66 (17.83%)	17 (10.00%)	12 (8.57%)	12,158 (12.01%)	895,038 (17.36%)
	Asian	39 (3.28%)	28 (3.09%)	10 (8.77%)	14 (3.78%)	10 (5.88%)	10 (7.14%)	4259 (4.20%)	309,043 (5.99%)
	American Indian or Alaska Native	12 (1.01%)	10 (1.10%)	0 (0%)	10 (2.70%)	0 (0%)	0 (0%)	657 (0.65%)	30,155 (0.59%)
	Native Hawaiian or Other Pacific Islander	10 (0.84%)	10 (1.10%)	10 (8.77%)	10 (2.70%)	0 (0%)	0 (0%)	735 (0.73%)	52,687 (1.02%)
	Other/Unknown Race	100 (8.40%)	72 (7.95%)	22 (19.29%)	35 (9.45%)	22 (12.93%)	22 (15.71%)	11,672 (11.53%)	1,073,199 (20.81%)
Diagnoses	Alcohol-related disorders	29 (2%)	22 (2%)	10 (9%)	10 (3%)	10 (6%)	10 (7%)	3047 (3%)	3969 (1%)
	Nicotine dependence	106 (9%)	86 (10%)	18 (16%)	24 (6%)	15 (9%)	12 (9%)	13,594 (13%)	17,373 (3%)

**Table 2:** Risk of adverse pregnancy outcomes (APOs) in patients with chronic inflammatory barrier diseases (CIBDs) treated with systemic therapies during pregnancy and control groups. Incidence (%) and absolute numbers of selected adverse pregnancy outcomes (APOs) are shown. Data are presented as percentage of affected pregnancies with corresponding event counts/total number of pregnancies per group. TNFi, tumor necrosis factor inhibitor; IL-23i, interleukin-23 inhibitor; MTX, methotrexate; CIBD, chronic inflammatory barrier disease; APO, adverse pregnancy outcome; Prg, pregnant. All rates presented are unadjusted cumulative incidence estimates. Adjusted comparative analyses are reported in Section 3.3 and Supplementary Table S8. Green shading indicates the lowest incidence and red shading indicates the highest incidence for each APO across all groups.

	TNFi % I Outcome/Total		TNFi (Without Certolizumab Pegol) % Ι Outcome/Total		MTX % Ι Outcome/Total		Azathioprine % Ι Outcome/Total		IL-23i % Ι Outcome/Total		Ustekinumab (IL-12/23i) % Ι Outcome/Total		Control 1: Prg + CIBD (Untreated) % Ι Outcome/Total		Control 2: All Pregnant Women % I Outcome/Total	
PRETERM LABOR	4.56	51/1119	3.97	34/857	0.00	0/100	4.62	15/325	6.10	≤10/164	7.35	≤10/136	2.23	2094/93,961	3.70	188,186/5,091,860
(PRE-)ECLAMPSIA	7.65	85/1111	6.92	59/853	9.90	≤10/101	4.46	14/314	6.71	11/164	7.41	≤10/135	2.57	2464/96,075	3.77	191,824/5,095,433
HYPERTENSION	10.70	114/1065	11.59	96/828	9.26	≤10/108	7.47	23/308	10.00	15/150	9.45	12/127	3.97	3739/94,252	5.58	283,540/5,083,051
PROTEINURIA	2.76	32/1159	2.72	24/884	0.00	0/110	2.86	≤10/350	5.99	≤10/167	7.30	≤10/137	0.84	839/99,705	1.14	58,673/5,147,486
GESTATIONAL DIABETES MELLITUS	3.86	42/1088	3.72	31/834	10.53	≤10/95	2.96	10/338	6.37	≤10/157	7.52	≤10/133	2.41	2288/94,811	4.04	204,374/5,065,265
ABORTION OR INTRAUTERINE DEATH	1.20	13/1084	1.44	12/832	0.00	0/99	2.98	≤10/336	6.29	≤10/159	7.58	≤10/132	2.20	2091/94,982	3.28	166,021/5,058,498
ANY APO	17.20	139/808	16.75	107/639	15.15	≤10/66	13.15	28/213	17.95	21/117	19.00	19/100	11.40	8879/76,126	16.63	786,741/7,731,295

## Data Availability

All relevant data are available within the main text, figures, and Supplementary Materials.

## Supplementary Materials

The following supporting information can be downloaded at: https://www.mdpi.com/article/10.3390/biom16081083/s1, Table S1: Pregnancy-Related Codes. ICD-10-CM, International Classification of Diseases, Tenth Revision, Clinical Modification; ICD-10-PCS, International Classification of Diseases, Tenth Revision, Procedure Coding System; LOINC, Logical Observation Identifiers Names and Codes; CPT, Current Procedural Terminology; Table S2: Definition of study and control groups according to underlying disease and medication exposure. IBD, inflammatory bowel disease; RA, rheumatoid arthritis; TNFi, tumor necrosis factor inhibitor; IL-23i, interleukin-23 inhibitor; csDMARD, conventional synthetic disease-modifying antirheumatic drug; Table S3: Adverse pregnancy outcomes (APOs) included in the analysis and corresponding ICD-10 codes. APOs were evaluated as a composite endpoint (“Any APO”) and as selected individual outcomes. ICD, International Classification of Diseases; Table S4: Sensitivity Analysis 1: Risk of adverse pregnancy outcomes (APOs) in patients with Inflammatory Bowel Disease (IBD) treated with systemic therapies during pregnancy and CIBD control group; Table S5: Sensitivity Analysis 2: Adverse pregnancy outcomes (APOs) among patients with chronic inflammatory barrier diseases (CIBD), restricted to pregnancies in adults aged 18 to 55 years; Table S6: Sensitivity Analysis 3: Adverse pregnancy outcomes (APOs) among patients with chronic inflammatory barrier diseases (CIBD), restricted to pregnancies with index event in 2019 or later; Table S7: Baseline characteristics of TNF inhibitor (TNFi) treated vs. chronic inflammatory barrier disease (CIBD) control patients before and after propensity-score matching (PSM). Values in parentheses and italics represent post-matching results; Table S8: Risk of adverse pregnancy outcomes (APOs) in patients with chronic inflammatory barrier disease (CIBD) treated with TNF inhibitors (TNFi) during pregnancy, compared to CIBD Control.

## Abbreviations

The following abbreviations are used in this manuscript:

APO	Adverse pregnancy outcome
CIBD	Chronic inflammatory barrier disease
CD	Crohn’s disease
csDMARD	Conventional synthetic disease-modifying antirheumatic drug
CPT	Current Procedural Terminology
CZP	Certolizumab pegol
EHR	Electronic health record
HR	Hazard ratio
IBD	Inflammatory bowel disease
ICD-10-CM	International Classification of Diseases, 10th Revision, Clinical Modification
ICD-10-PCS	International Classification of Diseases, 10th Revision, Procedure Coding System
IL	Interleukin
IL-12/23i	Interleukin-12/23 inhibitor
IL-23i	Interleukin-23 inhibitor
IMID	Immune-mediated inflammatory disease
MTX	Methotrexate
PSM	Propensity score matching
RA	Rheumatoid arthritis
RCT	Randomized controlled trial
RWE	Real-world evidence
TNF	Tumor necrosis factor
TNFi	Tumor necrosis factor inhibitor
UC	Ulcerative colitis
