# Supplementary material for: Risk of Adverse Pregnancy Outcomes in Patients with Non-Communicable, Chronic Inflammatory Barrier Diseases Under Systemic Treatment: A Large-Scale Retrospective Cohort Study
Source: Biomolecules. 2026 Jul 24;16(8):1083. doi: 10.3390/biom16081083 (PMC13511171; doi:10.3390/biom16081083)
Supplement: Supplementary file 1 [file biomolecules-16-01083-s001.zip › biomolecules-4411550-supplementary.pdf]

Supplementary Materials:

Code Type	Code	Exact Term / Description
ICD-10-CM	Z33.3	Pregnant state, gestational carrier
ICD-10-CM	Z33.1	Pregnant state, incidental
ICD-10-CM	Z33	Pregnant state
ICD-10-CM	Z34	Encounter for supervision of normal pregnancy
ICD-10-CM	Z3A	Weeks of gestation
ICD-10-CM	Z3A.0	Weeks of gestation of pregnancy, unspecified or less than 10 weeks
ICD-10-CM	Z3A.1	Weeks of gestation of pregnancy, weeks 10–19
ICD-10-CM	Z3A.2	Weeks of gestation of pregnancy, weeks 20–29
ICD-10-CM	Z3A.3	Weeks of gestation of pregnancy, weeks 30–39
ICD-10-CM	Z3A.4	Weeks of gestation of pregnancy, weeks 40 or greater
ICD-10-CM	Z36	Encounter for antenatal screening of mother
ICD-10-CM	Z32.01	Encounter for pregnancy test, result positive
ICD-10-CM	Z32.2	Encounter for childbirth instruction
ICD-10-CM	Z39	Encounter for maternal postpartum care and examination
ICD-10-CM	Z03.7	Encounter for suspected maternal and fetal conditions ruled out
ICD-10-CM	O09	Supervision of high-risk pregnancy
ICD-10-CM	O10–O16	Edema, proteinuria and hypertensive disorders in pregnancy, childbirth and the puerperium
ICD-10-CM	O20–O29	Other maternal disorders predominantly related to pregnancy
ICD-10-CM	O30–O48	Maternal care related to fetus and amniotic cavity and possible delivery problems
ICD-10-PCS	BY49	Fetus and Obstetrical / Ultrasonography / First Trimester, Single Fetus
ICD-10-PCS	BY4B	Fetus and Obstetrical / Ultrasonography / First Trimester, Multiple Gestation
ICD-10-PCS	BY48	Fetus and Obstetrical / Ultrasonography / Placenta
ICD-10-PCS	BY4C	Fetus and Obstetrical / Ultrasonography / Second Trimester, Single Fetus

ICD-10-PCS	BY4D	Fetus and Obstetrical / Ultrasonography / Second Trimester, Multiple Gestation
ICD-10-PCS	BY4F	Fetus and Obstetrical / Ultrasonography / Third Trimester, Single Fetus
ICD-10-PCS	BY4G	Fetus and Obstetrical / Ultrasonography / Third Trimester, Multiple Gestation
ICD-10-PCS	BY47	Fetus and Obstetrical / Ultrasonography / Fetal Umbilical Cord
ICD-10-PCS	BY	Fetus and Obstetrical
ICD-10-PCS	10	Pregnancy
LOINC	19080-1	Choriogonadotropin [Units/volume] in Serum or Plasma: > 25 mIU/mL
CPT	1010785	Diagnostic Ultrasound Procedures of the Pelvis Obstetrical

Table S1: Pregnancy-Related Codes. ICD-10-CM, International Classification of Diseases, Tenth Revision, Clinical Modification; ICD-10-PCS, International Classification of Diseases, Tenth Revision, Procedure Coding System; LOINC, Logical Observation Identifiers Names and Codes; CPT, Current Procedural Terminology.

Group	CIBD	Medication exposure
**Group 1**	Psoriasis, IBD, RA	TNFi (certolizumab pegol, golimumab, adalimumab, infliximab)
**Group 2**	Psoriasis, IBD, RA	TNFi (excluding certolizumab pegol)
**Group 3**	Psoriasis, IBD, RA	Methotrexate (csDMARD)
**Group 4**	Psoriasis, IBD, RA	Azathioprine (csDMARD)
**Group 5**	Psoriasis, IBD, RA	IL-23i (tildrakizumab, ustekinumab, risankizumab, guselkumab)
**Group 6**	Psoriasis, IBD, RA	Ustekinumab
**CIBD Control Group**	Psoriasis, IBD, RA	No exposure to above medications
**Control Group 2**	N/A (all pregnant women)	No exposure to above medications

Table S2: Definition of study and control groups according to underlying disease and medication exposure. IBD, inflammatory bowel disease; RA, rheumatoid arthritis; TNFi, tumor necrosis factor inhibitor; IL-23i, interleukin-23 inhibitor; csDMARD, conventional synthetic disease-modifying antirheumatic drug.

Category	Adverse Pregnancy Outcome	ICD Code
Any APO	Preterm labor	O60
	Pre-existing hypertension with pre-eclampsia	O11
	Gestational edema and proteinuria without hypertension	O12
	Gestational diabetes mellitus	O24.4
	Congenital eye malformations	Q13, Q14, Q15
	Spontaneous abortion	O03
	Intrauterine fetal death	O36.4
	Maternal urogenital tract infections	O23
	Unspecified maternal hypertension	O16
Selected APOs	Preterm labor	O60
	(Pre-)eclampsia	O11, O14, O15
	Hypertension	O13, O16
	Proteinuria	O12
	Gestational diabetes	O24.4
	Spontaneous abortion and/or intrauterine fetal death	O03 and O36.4

Table S3: Adverse pregnancy outcomes (APOs) included in the analysis and corresponding ICD-10 codes. APOs were evaluated as a composite endpoint ("Any APO") and as selected individual outcomes. ICD, International Classification of Diseases.

	TNF-I % I outcome /total		TNF-I (w/o CZP) % I outcome/total		MTX % I outcome/total		AZA % I outcome/total		IL-23-I % I outcome/total		USTE % I outcome/total		CONTROL 1: PRG + CID (without med) % I outcome/total	
Preterm labor	4.83	I 32/683	4.64	I 28/603	N = 10		5.26	I ≤ 10/190	8.55	I ≤ 10/117	9.62	I ≤ 10/104	2.84	I 843/ 29,728
(Pre)eclampsia	8.17	I 55/673	8.14	I 50/614	Cohort too small		5.50	I ≤ 10/182	8.33	I ≤ 10/120	9.52	I ≤ 10/105	2.81	I 858/ 30,444
Hypertension	11.37	I 74/651	12.46	I 74/594			6.42	I 12/ 187	11.82	I 13/ 110	12.37	I 12/97	4.25	I 1,276/ 30,016
Proteinuria	3.17	I 22/695	3.32	I 21/633			4.72	I ≤ 10/212	8.33	I ≤ 10/120	9.52	I ≤ 10/105	0.86	I 270/ 31,366
Gestational diabetes mellitus	4.07	I 27/663	4.30	I 26/605			5.13	I ≤ 10/ 195	8.62	I ≤ 10/116	9.90	I ≤ 10/101	2.53	I 765/ 30,217
Abort or intrauterine death	1.70	I 11/647	1.86	I 11/ 590			4.98	I ≤ 10/201	8.93	I ≤ 10/112	10.20	I ≤ 10/98	2.44	I 732/ 30,060
Any APO	17.73	I 86/485	18.16	I 81/446			12.31	I 16/130	20.69	I 18/87	22.67	I 17/75	12.49	I 3,127/25,037

Table S4: Sensitivity Analysis 1: Risk of adverse pregnancy outcomes (APOs) in patients with Inflammatory Bowel Disease (IBD) treated with systemic therapies during pregnancy and CIBD control group.

	TNF-I % I outcome /total		TNF-I (W/o CZP) % I outcome/total		MTX % I outcome/total		Azathioprine % I outcome/total		IL-23-I % I outcome/total		Ustekinumab % I outcome/total		CID Control % I outcome/total	
Preterm labor	4.59	I 50/1,090	4.09	I 34/831	0	I 0/73	5.62	I ≤ 10/178	6.54	I ≤ 10/153	8.26	I ≤ 10/121	2.15	I 2,242/104,252
(Pre)eclampsia	7.88	I 85/1,079	7.43	I 61/821	0	I 0/75	6.29	I ≤ 10/159	7.90	I 12/152	8.40	I ≤ 10/119	2.63	I 2,790/106,013
Hypertension	11.49	I 118/1,027	12.96	I 103/795	0	I 0/79	6.85	I ≤ 10/146	10.64	I 15/141	9.82	I 11/112	4.14	I 4,300/103,909
Proteinuria	3.10	I 35/1,131	3.28	I 28/854	0	I 0/82	5.68	I ≤ 10/176	6.37	I ≤ 10/157	8.13	I ≤ 10/123	0.86	I 944/110,002
Gestational Diabetes mellitus	3.48	I 37/1,064	3.47	I 28/808	12.66	I ≤ 10/79	5.78	I ≤ 10/173	6.80	I ≤ 10/147	8.48	I ≤ 10/118	2.39	I 2,506/104,753
Abort or intrauterine death	1.62	I 17/1,048	2.01	I 16/798	0	I 0/74	0	I 0/170	6.67	I ≤ 10/150	8.48	I ≤ 10/118	2.25	I 2,360/104,822
Any APO	17.41	I 133/764	17.44	I 105/602	19.23	I ≤ 10/52	10.19	I 11/108	21.05	I 24/114	22.22	I 20/90	11.30	I 9,589/84,892

Table S5: Sensitivity Analysis 2: Adverse pregnancy outcomes (APOs) among patients with chronic inflammatory barrier diseases (CIBD), restricted to pregnancies in adults aged 18 to 55 years.

	TNF-I % I outcome /total		TNF-I (W/o CZP) % I outcome/total		MTX % I outcome/total		AZA % I outcome/total		IL-23-I % I outcome/total		USTE % I outcome/total		CONTROL 1: PRG + CID (without med) % I outcome/total	
Preterm labor	4.17	I 31/744	4.01	I 22/549	0	I 0/34	4.01	I 12/299	6.99	I ≤ 10/143	8.26	I ≤ 10/121	2.40	I 309/12,868
(Pre)eclampsia	9.32	I 67/719	9.14	I 48/525	0	I 0/33	3.89	I 11/283	8.39	I 12/143	8.40	I ≤ 10/119	3.84	I 492/12,814
Hypertension	13.82	I 94/680	15.75	I 80/508	0	I 0/33	7.58	I 21/277	10.69	I 14/131	9.82	I 11/112	6.12	I 762/12,452
Proteinuria	2.49	I 19/762	2.88	I 16/555	0	I 0/37	3.12	I ≤ 10/321	6.80	I ≤ 10/147	8.13	I ≤ 10/123	1.26	I 167/13,231
Gestational diabetes mellitus	4.02	I 29/722	4.33	I 23/531	0	I 0/34	3.24	I ≤ 10/309	7.30	I ≤ 10/137	8.48	I ≤ 10/118	3.34	I 424/12,709
Abort or intrauterine death	2.00	I 14/701	2.53	I 13/514	0	I 0/35	3.26	I ≤ 10/307	7.09	I ≤ 10/141	8.48	I ≤ 10/118	3.29	I 413/12,546
Any APO	18.42	I 93/505	18.70	I 72/385	0	I 0/25	13.54	I 26/192	20.95	I 22/105	22.22	I 20/90	14.82	I 1,546/10,433

Table S6: Sensitivity Analysis 3: Adverse pregnancy outcomes (APOs) among patients with chronic inflammatory barrier diseases (CIBD), restricted to pregnancies with index event in 2019 or later.

Before matching, (After matching)		TNFi	CIBD Control	*p-value*	*Std diff.*
Demographics	**N (Total)**	1188<br>*(1188)*	101257<br>*(1188)*	—	—
	**Age (mean ± SD)**	31 ± 6.09<br>*(31 ± 6.09)*	34.9 ± 8.93<br>*(31 ± 6.16)*	< 0.0001<br>*(0.9413)*	0.5026<br>*(0.0030)*
	**White (2106-3)**	886 (74.58%)<br>*(886 (74.60%))*	71,775 (70.88%)<br>*(897 (75.51%))*	0.0053<br>*(0.6021)*	0.0830<br>*(0.0214)*
	**Black or African American (2054-5)**	145 (12.21%)<br>*(145 (12.21%))*	12,158 (12.01%)<br>*(137 (11.53%))*	0.8344<br>*(0.6118)*	0.0061<br>*(0.0208)*
	**Hispanic or Latino (2135-2)**	112 (9.43%)<br>*(112 (9.43%))*	11,447 (11.31%)<br>*(114 (9.60%))*	0.0420<br>*(0.8888)*	0.0616<br>*(0.0057)*
Diagnoses	**Overweight (E66)**	243 (20.46%)<br>*(243 (20.46%))*	20,870 (20.61%)<br>*(224 (18.86%))*	0.8946<br>*(0.3267)*	0.0039<br>*(0.0403)*
	**Hypertensive diseases (I10–I1A)**	113 (9.51%)<br>*(113 (9.51%))*	14,695 (14.51%)<br>*(115 (9.68%))*	< 0.0001<br>*(0.8892)*	0.1543<br>*(0.0057)*
	**Socioeconomic risk (Z55–Z65)**	67 (5.64%)<br>*(67 (5.64%))*	5,221 (5.16%)<br>*(63 (5.30%))*	0.4539<br>*(0.7182)*	0.0214<br>*(0.0148)*
	**Nicotine dependence (F17)**	106 (8.92%)<br>*(106 (8.92%))*	13,594 (13.43%)<br>*(102 (8.59%))*	< 0.0001<br>*(0.7715)*	0.1433<br>*(0.0119)*
	**Alcohol-related disorders (F10)**	29 (2.44%)<br>*(29 (2.44%))*	3,048 (3.01%)<br>*(23 (1.94%))*	0.2532<br>*(0.4002)*	0.0350<br>*(0.0345)*
	**Diabetes mellitus (E08–E13)**	52 (4.38%)<br>*(52 (4.38%))*	7,112 (7.02%)<br>*(44 (3.70%))*	0.0004<br>*(0.4046)*	0.1143<br>*(0.0342)*

	**Chronic kidney disease (N18)**	14 (1.18%)<br>*(14 (1.18%))*	1,746 (1.724%)<br>*(11 (0.93%))*	0.1500<br>*(0.5464)*	0.0457<br>*(0.0248)*
Medications	**Adrenal corticosteroids (HS050)**	971 (81.73%)<br>*(971 (81.73%))*	54,630 (53.95%)<br>*(968 (81.48%))*	< 0.0001<br>*(0.8738)*	0.6230<br>*(0.0065)*
	**Gastric medications (GA900)**	762 (64.14%)<br>*(762 (64.14%))*	37,994 (37.52%)<br>*(762 (64.14%))*	< 0.0001<br>*(1.0000)*	0.5524<br>*(< 0.0001)*
	**Sulfasalazine (RxNorm 9524)**	124 (10.44%)<br>*(124 (10.44%))*	1,889 (1.866%)<br>*(118 (9.93%))*	< 0.0001<br>*(0.6840)*	0.3626<br>*(0.0167)*

Table S7: Baseline characteristics of TNF inhibitor (TNFi) treated vs. chronic inflammatory barrier disease (CIBD) control patients before and after propensity-score matching (PSM). Values in parentheses and italics represent post-matching results.

	TNFi		CIBD control		risk difference (95% ci)	HR (95% CI)	p ($a_{adj} = 0.0071$)
	% ι outcome/total		% ι outcome/total				
**Preterm labor**	4.56	I 51/ 1119	2.75	I 30/ 1091	1.81% (0.25%,3.37%)	1.61 (1.02,2.52)	0.0372
**(Pre)eclampsia**	7.65	I 85/ 1111	3.67	I 41/ 1118	3.98% (2.07%,5.90%)	2.07 (1.43,3.01)	< 0.0001 †
**Hypertension**	10.70	I 114/ 1065	5.40	I 59/ 1092	5.30% (3.01%,7.59%)	1.97 (1.44,2.70)	< 0.0001 †
**Proteinuria**	2.76	I 32/ 1159	1.11	I 13/ 1168	1.65% (0.53%,2.77%)	2.40 (1.26,4.57)	0.0060 †
**Gestational diabetes mellitus**	3.86	I 42/ 1088	2.83	I 32/ 1131	1.03% (-0.47%,2.53%)	1.31 (0.83,2.08)	0.2464
**Abortion or intrauterine death**	1.20	I 13/ 1084	2.93	I 33/ 1128	-1.73% (-2.90%,-0.55%)	0.39 (0.21,0.74)	0.0029 †
**Any apo**	17.20	I 139/ 808	14.86	I 134/ 902	2.35% (-1.14%,5.83%)	1.12 (0.89,1.42)	0.3434

p-values are Bonferroni-adjusted with an alpha level of 0.0071. † indicates statistical significance ($p < 0.0071$)

Table S8: Risk of adverse pregnancy outcomes (APOs) in patients with chronic inflammatory barrier disease (CIBD) treated with TNF inhibitors (TNFi) during pregnancy, compared to CIBD Control.
